# Endoscopic direct diverticulitis therapy for acute uncomplicated diverticulitis

**DOI:** 10.1055/a-2587-4966

**Published:** 2025-05-14

**Authors:** Jian-Zhen Ren, Jun Cai, Bo Li, Guang Yang, Su-Huan Liao, Jia-Kuan Chen, Yi-Tian Guo, Yang-Bor Lu, Si-Lin Huang

**Affiliations:** 1 Department of Gastroenterology, South China Hospital, Medical School, Shenzhen University, Shenzhen, China; 2 Department of Radiology, South China Hospital, Medical School, Shenzhen University, Shenzhen, China; 3 Department of Digestive Disease, Xiamen Chang Gung Hospital, Hua Qiao University, Xiamen, China; 4 Endoscopy Center, Xiamen Chang Gung Hospital, Hua Qiao University, Xiamen, China

## Abstract

**Background:**

The global prevalence of acute uncomplicated diverticulitis is increasing, and patient care for this condition is experiencing a paradigm shift. This study evaluated the diagnostic and therapeutic value of endoscopic direct diverticulitis therapy (EDDT) using cholangioscope-assisted colonoscopy for acute uncomplicated diverticulitis.

**Methods:**

Patients with computed tomography-confirmed acute uncomplicated diverticulitis (Hinchey stage Ia or Ib) who underwent EDDT between July 2023 and December 2024 were included. The technical success rate of EDDT, procedure time, endoscopic findings, symptom resolution, and recurrence were documented.

**Results:**

12 patients (mean age 45.2 [SD 10.1] years, 10 males) underwent EDDT. Technical success was achieved in all 12 patients (100%). Cholangioscope-assisted colonoscopy revealed fecaliths with pus in seven patients, copious purulent collection in four, edematous inflammatory mucosa in five, and bleeding in one. Corresponding treatments were applied without complications. Abdominal pain resolved immediately after EDDT, and inflammatory parameters returned to normal. No recurrences were reported over a mean follow-up of 4 months (range 1–10 months).

**Conclusions:**

EDDT utilizing cholangioscope-assisted colonoscopy was a feasible alternative treatment for acute uncomplicated diverticulitis, offering diagnostic and therapeutic advantages through direct visualization. Although preliminary results are promising, further studies with larger cohorts are warranted to confirm its efficacy and long-term outcomes.

## Graphical Abstract

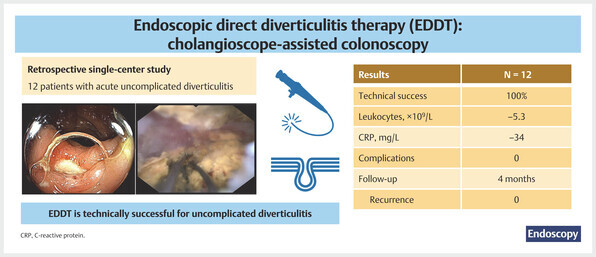

## Introduction

The prevalence of acute uncomplicated diverticulitis is rising worldwide, partly due to advancements in imaging techniques that enable more accurate diagnoses and partly because of lifestyle changes 1 . Several guidelines have recommended the conditional use of antibiotics rather than routine administration 2 . However, the current guidelines predominantly referred to left-sided diverticulitis in a common type of Western population, and a higher proportion of patients undergoing nonantibiotic approaches have required elective surgery compared with those receiving antibiotics 3 . Many centers continue to prescribe antibiotics in alignment with local protocols 4 due to the uncertain etiologies of diverticulitis.

To address this clinical challenge, we previously introduced the first endoscopic direct diverticulitis therapy (EDDT) 5 , which allows for lesion visualization and tailored management using a cholangioscope initially designed for biliary tract inspection. We achieved preliminary satisfactory clinical outcomes without recurrence by utilizing this intraluminal scope. This pilot endoscopic approach extends the capabilities of conservative therapy by incorporating active intervention and guides subsequent management decisions, including antibiotic use. In this study, we aimed to investigate the diagnostic and therapeutic value of using cholangioscope-assisted colonoscopy for EDDT in acute uncomplicated diverticulitis.

## Method

### Study design and protocol

The medical charts of adult patients diagnosed with acute uncomplicated diverticulitis and admitted to the South China Hospital of Shenzhen University between July 2023 and December 2024 were reviewed for eligibility. The study was approved by the Ethics Committee of South China Hospital of Shenzhen University on July 23, 2024 (Approval No. HNLS20240723002-A). The inclusion criteria were individuals aged ≥18 years with a first-time admission for acute uncomplicated diverticulitis, verified by computed tomography (CT) as modified Hinchey classification stage Ia or Ib, following the criteria applied in the DIABLO trial 6 . Exclusion criteria included patients with previous diverticulitis, higher modified Hinchey stages, other abdominal infections (e.g. liver abscess, cystitis, and pancreatitis), a history of gastrointestinal perforation or inflammatory bowel disease, and those unwilling to undergo endoscopic therapy or unsuitable for EDDT due to known perforation risk (e.g. immunocompromised status, use of corticosteroid and/or opioid analgesics) 7 8 . Patients who met the criteria were presented with two treatment options – antibiotics alone or antibiotics combined with endoscopic therapy. Those who elected to undergo EDDT were enrolled. All authors had secured data-use agreements with the medical database custodians, allowing them controlled access to individual-level patient data. Dedicated informed consent specific to EDDT, including a thorough explanation of the risks, benefits, and alternatives to EDDT, was obtained from each patient who opted for endoscopic treatment.

### Pre-procedure preparation

All patients underwent routine and thorough bowel preparation using three packs of polyethylene glycol powder, each mixed with water to obtain 1 L of electrolyte solution. They were instructed to consume the solution within 4 hours before the procedure.

### Description of the technique

Demonstration of endoscopic direct diverticulitis therapy performed for colonic diverticulitis.Video 1

**Fig. 1:**
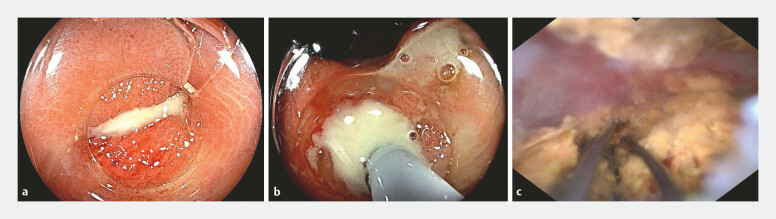
The application of endoscopic direct diverticulitis therapy. **a** Colonoscopic inspection of the edematous diverticular orifice. **b** Cholangioscopic view of the intradiverticulum, with copious pus collection. **c** Cholangioscopic view of the fragmentation and extraction of fecaliths using a disposable retrieval basket.

**Fig. 2:**
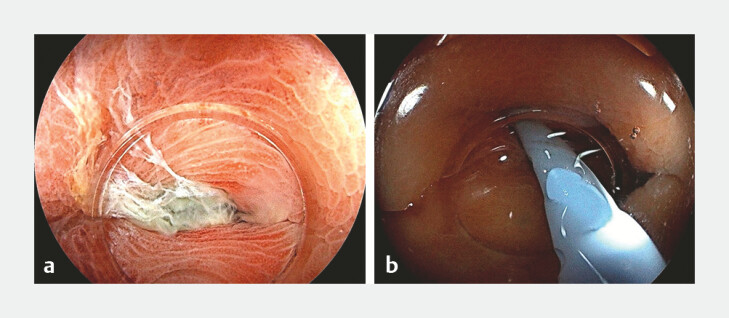
Colonoscopic view. **a** Spontaneous purulent exudate from the congested orifice. **b** Pancreatic duct stent deployment.

After adequate preparation, patients were placed in the left horizontal position and received general anesthesia (propofol and fentanyl). Initially, a colonoscope (EC-601WM/EC-760R-V/M; Fujifilm, Tokyo, Japan) with carbon dioxide insufflation equipped with a transparent cap was introduced to locate the entrance of the diverticulum and evaluate signs of inflammation, such as mucosal congestion, edema, or discharge ( Fig. 1 **a** ). Following careful observation of the diverticular orifice, a cholangioscope (eyeMax, 9 Fr; Micro-Tech, Nanjing, China) was introduced through the working channel of the colonoscope and navigated into the diverticulum ( Fig. 1 **b** ), implementing EDDT ( Video 1 ). The cholangioscope has various tools, facilitating interventions such as clipping, basket retrieval, or lithotripsy apparatus ( Fig. 1 **c** ). This therapy included repeated lavage, and deployment of a pancreatic duct stent (7-Fr, PBD-234–0708; Olympus, Tokyo, Japan) to ensure drainage when patients presented with spontaneous expulsion of purulent exudate from the orifice ( Fig. 2 ). Technical success was defined as the successful intubation of the diverticulum. Clinical variables, endoscopic manifestation, and laboratory parameters before and 72 hours after the procedure were also documented.

### Post-procedure care and follow-up

Patients received antibiotics orally or intravenously, as indicated by the endoscopic findings, and could consume a soft diet on the same day. After the procedure, patients remained in hospital for at least 24 hours and were discharged thereafter if no symptoms suggestive of adverse effects, including perforation, had developed. Follow-up appointments were scheduled for 1 week post-intervention, except for those experiencing worsening signs and symptoms. A follow-up CT was planned for all patients to assess the resolution of inflammation (e.g. changes in colonic wall thickness). Those with stent deployment were informed about the need for stent removal via colonoscopy if spontaneous discharge did not occur within the follow-up time.

### Statistical analysis

This pilot study did not require inferential statistics. Descriptive statistics were used to present the overall study population. Data were statistically analyzed using Statistical Analysis System software version 9.4 (SAS Institute, Cary, North Carolina, USA). The mean (SD) was calculated for quantitative variables, and frequency (%) was used for qualitative variables.

## Results

A total of 12 patients (10 males) with a mean age of 45.2 (SD 10.1) years were included in the study. All procedures were performed by two experienced endoscopists to ensure consistency in technique and minimize interoperator variability during this preliminary stage of the study. Clinical symptoms are detailed in Table 1 . Notably, 11 of the 12 patients presented with right-sided diverticulitis, a subtype more common in non-Western populations. Additionally, all patients had elevated inflammatory markers (e.g. leukocytosis and/or elevated C-reactive protein), indicating an infectious component, and a course of broad-spectrum antibiotics was administered according to the Chinese guidelines for intra-abdominal infection (IAI). Eight of 12 patients (66.7%) presented with Hinchey stage Ib, six (50.0%) had involvement of the ascending colon, and the mean maximum wall thickness was 15.4 (4.6) mm. Three patients underwent follow-up CT, revealing a decrease in maximum wall thickness from 20.7 (5.0) mm to 13 (3.5) mm. The remaining patients did not undergo CT due to significant clinical improvement. Technical success was achieved in all patients. The mean procedure time, defined as the duration from the beginning of colonoscopy to the end of treatment, was 68.5 (SD 33.9) minutes.

**Table:** **Table 1** Baseline characteristics of patients receiving endoscopic direct diverticulitis therapy (n = 12).

Clinical characteristics	
Age, mean (SD) [range], years	45.2 (10.1) [32.0–60.0]
Sex, male, n (%)	10 (83.3)
Symptoms, n (%)	
Abdominal pain	6 (50.0)
Right lower abdomen pain	4 (33.3)
Left lower abdomen pain	1 (8.3)
Tarry stool	1 (8.3)
CT manifestation	
Hinchey stage, n (%)	
Ia	4 (33.3)
Ib	8 (66.7)
Multiple diverticula, n (%)	7 (58.3)
Involved segments, n (%)	
Cecum	5 (41.7)
Ascending colon	6 (50.3)
Transverse colon	0 (0.0)
Descending colon	0 (0.0)
Sigmoid colon	1 (8.3)
Maximum wall thickness, mean (SD) [range], mm	15.4 (4.6) [9–26]
Laboratory characteristics	
Leukocyte, mean (SD) [range], ×10 ^9^ /L	10.8 (4.4) [7.4–13.5]
Leukocytosis ^1^ , n (%)	5 (41.7)
Serum CRP, mean (SD) [range], mg/L	47.2 (37.8) [0.2–103.6]
Elevated serum CRP ^2^ , n (%)	9 (75.0)
Procedure time ^3^ , mean (SD) [range], minutes	68.5 (33.9) [10.0–107.0]
Follow-up time, mean (SD) [range], months	4.0 (3.1) [0.3–10.0]
CT, computed tomography; CRP, C-reactive protein; EDDT, endoscopic direct diverticulitis therapy. ^1^ Defined as >10 × 10 ^9^ /L. ^2^ Defined as >5 mg/L. ^3^ Defined as the duration from the beginning of colonoscopy to the end of treatment.	

Cholangioscope-assisted colonoscopy findings ( Table 2 ) revealed fecaliths with pus in seven patients, copious purulent collection in four patients, edematous inflammatory mucosa in five, and bleeding in one. Corresponding treatments were tailored and applied, including irrigation with metronidazole and sodium chloride, fecalith removal, and clipping. A stent was deployed in three patients and spontaneously dislodged 1 week after the procedure. The mean hospital stay after intervention was 4 (1.6) days.

**Table:** **Table 2** Endoscopic characteristics and outcome of endoscopic direct diverticulitis therapy (n = 12).

Endoscopic manifestations	
Colonoscopic view of diverticular orifice ^1^ , n (%)	
Congestion and edema of the diverticular orifice	10 (83.3)
Fecaliths impaction	2 (16.7)
Pus exudate	9 (75)
Cholangioscopic view of intradiverticular content, n (%)	
Fecaliths with pus	7 (58.3)
Copious purulent collection	4 (33.3)
Edematous and inflammatory mucosa	5 (41.7)
Bleeding lesion	1 (8.3)
Laboratory parameters 72 hours after EDDT, mean (SD) [range]	
Leukocyte, ×10 ^9^ /L	6.1 (2.1) [3.4–10.6]
Change from preoperative leukocyte, ×10 ^9^ /L	–5.3 (2.7) [–9.3 to –0.4]
Serum CRP, mg/L	17.7 (23.4) [0.5–62.2]
Change from preoperative CRP, mg/L	–33.9 (30.2) [–86.3 to +0.3]
Hospitalization stay, day	4.0 (1.6) [1.0–7.0]
CRP, C-reactive protein; EDDT, endoscopic direct diverticulitis therapy. ^1^ Some patients exhibited more than one finding, so the total number of manifestations exceeds the total number of patients.	

Patient-reported abdominal pain resolved after EDDT in all patients. Leukocyte and C-reactive protein levels gradually returned to normal limits following the procedure ( Table 2 ). There were no complications during or after the procedure, and no recurrences were reported over a mean follow-up period of 4.0 months (range 1–10 months).

## Discussion

Diverticulitis is caused by micro- or macroscopic perforations in the diverticular wall due to increased intraluminal pressures or inspissated food matter, leading to inflammation, ischemia, and focal necrosis 9 . Contemporary approaches advocate less aggressive medical and surgical management of acute diverticulitis 10 . Driven by this trend and advances in endoscopic technology, we developed a novel EDDT method that aligns with this strategy and provides individualized and precise therapy for diverticulitis 5 11 12 . Our cholangioscope-assisted colonoscopy enables direct lesion assessment by intubating the diverticulum, allowing immediate interventions, including fecalith retrieval, intraluminal decompression, lesion lavage, hemostasis, biopsy, and stent deployment. This approach tailors treatment efficiently to the individual’s condition. The current guidelines predominantly address left-sided colonic diverticulitis, typically seen in the Western population 13 14 . These recommendations may not fully apply to right-sided disease, a pattern more common in non-Western groups such as our study cohort, with varied ethnic backgrounds and possibly different underlying pathophysiological mechanisms. These differences can influence key management decisions, including whether to administer antibiotics. In such cases, EDDT functions as a therapeutic platform with integrated diagnostic capabilities. Endoscopic evaluation allows clinicians to better stratify disease severity, assess the need for antibiotics, and tailor treatment strategies to optimize patient outcomes.

Our EDDT method, with its customized therapeutic features, also enhances diagnostic clarity. CT has high sensitivity and specificity in diagnosing acute diverticulitis and is the first-line investigation in suspected cases. A proper classification system facilitates effective communication among physicians of different specialties and guides corresponding clinical management. Consequently, numerous classifications and modifications have been proposed to describe the variety of diverticular diseases 15 ; however, despite their varied emphases with unique strengths and limitations, none has been universally adopted or proven superior as a diagnostic tool 16 . The EDDT method introduces the capability of delineating detailed diverticular lesions exclusively through endoscopic modalities, enabling the endoscopic cataloging of diverticulitis. This addresses the challenge of integrating both diagnostic and therapeutic components. In three cases classified as Hinchey Ib diverticulitis, EDDT endoscopically revealed diverticulitis with fecaliths that were undetectable on diagnostic CT. Such clinical manifestations of uncomplicated fecalith infections have not been included in any current classification systems based on clinical and radiological findings, which may have potential inherent limitations 14 . Therefore, this advancement in real-time visualization could improve diagnostic accuracy and facilitate the triage of acute diverticulitis. It offers the possibility of developing classifications incorporating endoscopic findings to better link disease presentations with underlying mechanisms while guiding corresponding management and prognosis prediction.

The American Gastroenterological Association guidelines recommend delaying colonoscopy for 6 weeks following an acute episode of diverticulitis to mitigate perforation risk 13 . However, evidence supporting this precaution remains limited. Bar-Meir and colleagues concluded that early colonoscopy for confirmed acute colonic diverticulitis is both safe and feasible, based on findings from their prospective study as well as a randomized controlled trial 17 18 . Moreover, our study incorporated the following safety measures. First, our study focused on CT-confirmed uncomplicated diverticulitis, which typically has a lower risk of serious complications. Second, our cohort excluded patients with complicating risk factors such as immunosuppression. Third, the procedures employed carbon dioxide insufflation, an ultrathin high-resolution cholangioscope with minimal mechanical force, and were performed by experienced endoscopists. Taken together, previous studies, implemented safety measures, and the result of the pilot study suggest that EDDT is a safe therapeutic option for selected patients, though larger studies are needed to confirm these results.

We acknowledge several limitations in our study. First, as a pioneering endoscopic technique, EDDT has not been characterized and validated in the broader clinical context because of its innovation. Moreover, a small cohort size and the absence of a control group preclude definitive conclusions regarding overall effectiveness. Future research employing comparative study designs is necessary to further validate these findings. In addition, the unknown longer-term outcomes limit the generalizability of the result. While our endoscopic technique has yielded encouraging short-term results – with no recurrence observed during a 4-month mean follow-up – it demonstrates potential as an effective treatment for acute uncomplicated diverticulitis. Previous studies have indicated that a maximum thickness of the affected segment of >15 mm is associated with a sixfold increased risk of recurrence within 90 days 19 . In our study, five patients with a maximum thickness >15 mm were followed for over 90 days without recurrence. Similarly, eight patients classified as Hinchey Ib experienced no recurrence within a mean follow-up of 3 months. We are continuing to collect extended follow-up data to document enduring efficacy and to monitor for any possible late-onset complications. Future research should investigate how differing criteria might impact treatment strategies and outcomes. While these initial results are promising, further research with large cohorts and control groups is necessary to establish the efficacy of this innovative approach for uncomplicated acute diverticulitis.

In conclusion, EDDT utilizing cholangioscope-assisted colonoscopy in diverticular diseases may serve as a promising alternative treatment. The direct visualization provided by colonoscopy offers both diagnostic and therapeutic benefits, potentially enhancing patient care. Initially designed for biliary diseases, the advantages of this technique open the avenues for application to other conditions that benefit from endoscopic inspection, such as its previous use in uncomplicated appendicitis 20 . Our study demonstrates that these advantages can be broadly leveraged to treat uncomplicated diverticulitis.
